# MicroRNA Prediction Using a Fixed-Order Markov Model Based on the Secondary Structure Pattern

**DOI:** 10.1371/journal.pone.0048236

**Published:** 2012-10-30

**Authors:** Wei Shen, Ming Chen, Guo Wei, Yan Li

**Affiliations:** 1 Medical Research Center, Southwest Hospital, Third Military Medical University, Chongqing, China; 2 Bioinformatics Laboratory, Chongqing Key Laboratory for Disease Proteomics, Chongqing, China; Memorial Sloan Kettering Cancer Center, United States of America

## Abstract

Predicting miRNAs is an arduous task, due to the diversity of the precursors and complexity of enzyme processes. Although several prediction approaches have reached impressive performances, few of them could achieve a full-function recognition of mature miRNA directly from the candidate hairpins across species. Therefore, researchers continue to seek a more powerful model close to biological recognition to miRNA structure. In this report, we describe a novel miRNA prediction algorithm, known as FOMmiR, using a fixed-order Markov model based on the secondary structural pattern. For a training dataset containing 809 human pre-miRNAs and 6441 human pseudo-miRNA hairpins, the model’s parameters were defined and evaluated. The results showed that FOMmiR reached 91% accuracy on the human dataset through 5-fold cross-validation. Moreover, for the independent test datasets, the FOMmiR presented an outstanding prediction in human and other species including vertebrates, Drosophila, worms and viruses, even plants, in contrast to the well-known algorithms and models. Especially, the FOMmiR was not only able to distinguish the miRNA precursors from the hairpins, but also locate the position and strand of the mature miRNA. Therefore, this study provides a new generation of miRNA prediction algorithm, which successfully realizes a full-function recognition of the mature miRNAs directly from the hairpin sequences. And it presents a new understanding of the biological recognition based on the strongest signal’s location detected by FOMmiR, which might be closely associated with the enzyme cleavage mechanism during the miRNA maturation.

## Introduction

MicroRNAs (miRNAs) are ∼22-nucleotide RNAs derived from pri-miRNA transcripts through two important enzyme processes, where the first process is recognized and cut by Drosha and DGCR8 for pre-miRNA formation from pri-mRNA, and the second is by Dicer for miRNA maturation from pre-miRNA [1], [2], but the recognition mechanism is still obscure [2]–[5]. Although many miRNAs have been identified in some species by experimental method, it is believed that there are still a large number of miRNAs uncovered, including those with low expression or in other species [6], [7]. Therefore, computational prediction is regarded as a rapid and effective way to solve these problems in contrast to the hard experimental work, however, the diversity of the precursors and complexity of enzyme processes bring challenge for computational approaches to distinguish the real miRNAs from the pseudo-miRNA hairpins with similar stem-loops.

To date, there are mainly four kinds of computational approaches have been tried [8]: (1) A homology-based approach, such as miRNAlign [9], aligns the secondary structure of pre-miRNAs to detect miRNAs. (2) A filter-based approach, such as MIRScan [10] and MiRSeeker [11], picks out pre-miRNAs from an initial set of candidate stem-loops based on GC content, minimum free energy (MFE) and structural filters. (3) A target-centered approach depends on the highly conserved motifs in 3′-UTRs [12]. (4) Machine learning approaches include support vector machine (SVM), hidden Markov model (HMM) and naïve Bayes classifier (NBC), such as Triplet-SVM [6], MiPred [13], miRank [14], CID-miRNA [15], HHMMiR [16], CSHMM [17] and MatureBayes [18]. However, the first three approaches are poor to identify new miRNAs across species lack of homologies. Although the machine leaning approaches achieve satisfactory performance in several species, they are generally limited into a single-function prediction, for instance, either only predicting precursors from hairpins [6], [13]–[17] or miRNAs from precursors [18].

In this study, based on the secondary structure pattern of miRNA precursors, we try to find out a common structural feature associated with miRNA formation, and describe a new miRNA predictor by using a fixed-order Markov model in order to realize a full-function recognition of mature miRNA directly from the sequence segments with similar stem-loop hairpin across species.

## Materials and Methods

### Data Preparation

The sequences of miRNAs companied with their precursors were downloaded from miRBase database (release 16) [19], [20], containing 1046 sequences from human, 6746 from vertebrates, 580 from worms, 235 from viruses and 3052 from plants. Among them, 809 human miRNA sequences were randomly selected out as the positive training set (D1), the remaining 237 human sequences and all of those from other species as positive test set. On the other hand, there were 8494 human pseudo precursors and 754 ncRNAs obtained from microPred website [6], [21], where 5890 pseudo ones and 551 ncRNAs were randomly selected out as negative training set (D2), and the remaining as negative test set. Based on these data, the model’s parameters would be trained only in part of human sequences (D1 and D2), but be estimated in human itself and all other species.

### Construction of the Stem-bulge-gap Notation

**Figure 1:**
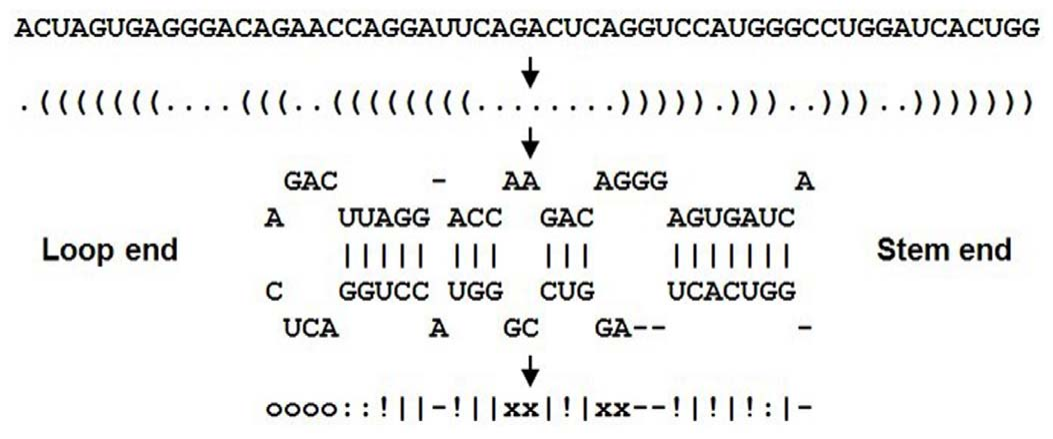
Illustration of the construction of the stem-bulge-gap notation. In the stem-bulge-gap notation at the bottom line, the symbols of ‘|’, ‘!’ and ‘:’ represent respectively the base pair of ‘CG’, ‘AU’ and ‘GU’, the symbols of ‘o’, ‘x’ and ‘-’ represent respectively the loop, bulge and gap. In the asymmetric bulges, the symmetric part is indicated with ‘x’ and the asymmetric part with ‘-’.

For this model study, we established a stem-bulge-gap notation to describe the secondary structure of hairpin. Figure 1 illustrated the construction process of the notation, at first, the dot-bracket notation was produced by RNAfold [22], [23], then converted to a stem-loop structure and finally converted into the stem-bulge-gap notation. Moreover, to avoid the noise from the stem-branches, we appointed the longest stem as the main stem, and treated other stem-branches into loops, bulges or gaps.

### The Establishment of a Fixed-order Markov Model for miRNA Recognition

A modified fixed-order Markov model was employed to explore the secondary structure pattern of miRNA on the stem-bulge-gap notation. According to the style of Begleiter [24] and Shmilovici [25], we let 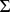 be a finite alphabet of size 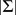. In the case of this paper 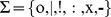 and 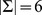. To consider a sequence 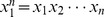 where 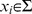 was the symbol at the position 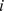, with 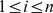 in the sequence and 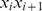 was the concatenation of 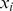 and 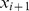. Based on the training set 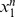, a model parameter 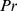 was assigned as the probability of the next symbol given the position and previous context. For a context 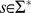 where the 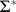 represents a fixed length of context set, the learner generated a conditional probability distribution 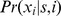 for each symbol 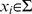. For variable-order Markov (VOM) model estimating conditional distribution of the 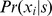 with a variant context length 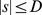, we proposed the conditional distribution with a fixed length 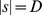, as a special case of the VOM model.

**Figure 2:**
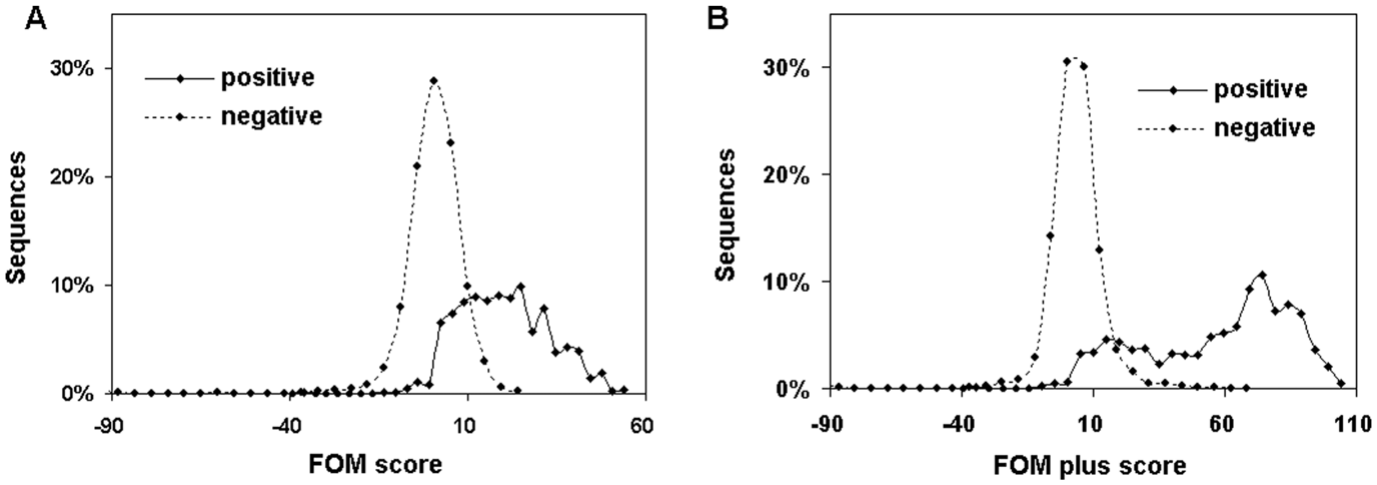
Distribution of the signal scores in positive and negative datasets.

**Figure 3:**
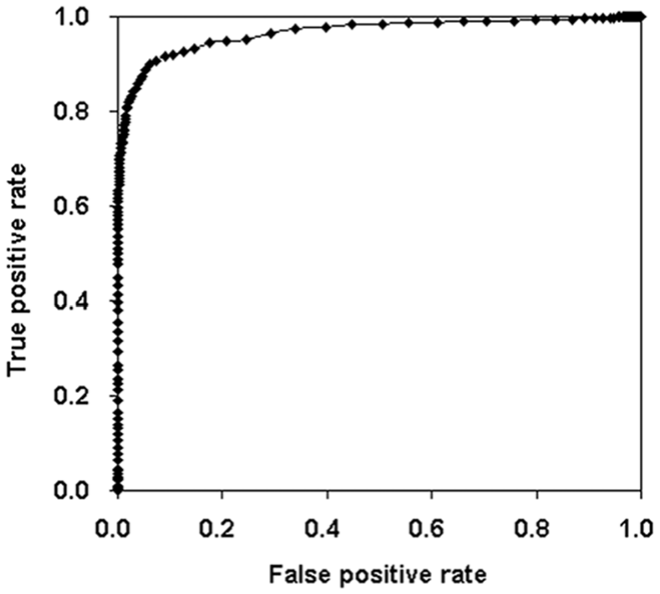
Receiver Operating Characteristic Curve of FOMmiR predictor.

To calculate the probability of the model, the count 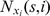 denoted the number of occurrences in which symbol 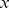 in position 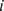 following context 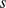 in the training sequence. The conditional probability was defined as
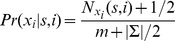
Where 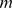 denoted the number of the training sequences. Once the conditional probability distribution was estimated, the probability of a sequence for a given model could be calculated by



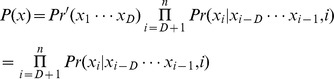
Where 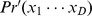 was the occurrence probability of the initial context, and we let it be 1. To distinguish pre-miRNAs from other hairpin sequences, a True model was constructed to represent true pre-miRNA and a False model for pseudo pre-miRNA. Then each stem-bulge-gap sequence 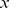 was scored by:



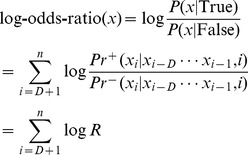



To handle events in different level of counts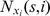, the calculation of 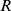 was defined asM
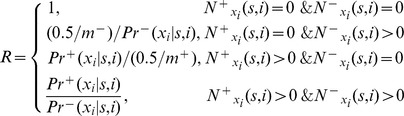



Another similar FOM model were used for mature miRNA strand identification. 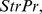 like 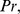 was assigned as the probability of strands given the previous context of stem-bulge-gap sequence and position.
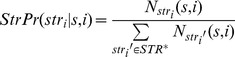
Where 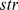 was a strand symbol, 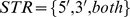 was alphabet of strands and 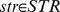, the count 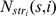 denoted the number of occurrences in which strand symbol 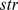 in position 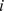 following context 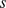. And the strand probabilities of a stem-bulge-gap sequence were calculated by



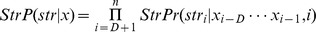



The 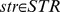 with maximum value of 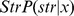 would be the strand of the sequence 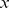.

### Feature Selection

In this study, thirty-six structure features from the previous studies were concerned about as well [21], [26]–[28]. Out of them, only three MFE-related features (MFEI1 [27] MFEI2 [28] MFEI4 [21]) were found be helpful to improve performance of FOM in certain level. Based on a binary logistic regression analysis, the coefficients (MFEI1: −0.209, MFEI2: 0.034, MFEI4: 1.679 and Const: −13.686) were adopted.

**Table 1:** The performances of pre-miRNA prediction.

Method	Year	Algorithm	Sen	Spe	Acc
Triplet-SVM	2005	Support vectormachine	72.15%	91.09%	89.62%
MiPred	2007	Random Forest	93.25%	6.59%	13.41%
CIDmiRNA	2008	Stochastic contextfree grammar	75.95%	96.29%	94.71%
CSHMM	2010	Context sensitive HMM	88.19%	71.46%	72.77%
FOMmiR	2012	Fixed order Markovmodel	89.45%	91.27%	91.13%

### Pipeline for the Prediction of miRNA

According to the above model definition, we constructed a miRNA predictor with the pipeline:

#### (1) Data preparation

All the hairpins were converted into the stem-bulge-gap notation for the model computation. The 24 bp stem-bulge-gap segments covering the mature miRNA in precursors of D1 dataset, and the same size segments sliding with 1 bp step size in pseudo miRNA precursors of D2 were used for calculating model parameters.

**Table 2:** Comparison of sensitivity across different species.

Method	Vertebrates(6746)	Plants(3052)	Drosophila(1205)	Worms(580)	Viruses(235)
Triplet-SVM	75.26%	65.27%	85.39%	85.00%	65.11%
MiPred	92.48%	47.02%	93.94%	95.52%	96.60%
CIDmiRNA	75.85%	73.23%	85.81%	86.90%	70.64%
CSHMM	93.60%	91.43%	95.68%	97.76%	91.06%
FOMmiR	91.76%	93.55%	97.18%	97.07%	89.79%

#### (2) Model training

A set of continuous FOM scores were calculated in a window size of 24 bp sliding on each hairpin from loop to tail with 1 bp step size, and three MFE-related features were added into FOM score as FOM plus score (FOM plus score  =  FOM score+50×Feature score) for improving the signal. After that, the best FOM plus score was screen out from the first peak followed by a valley of at least 5 bp size. Then one segment with the best score was screened out in each hairpin for judgment, meanwhile, the strand information (5′, 3′ or both) where miRNA located was collected as well. For the training dataset, a threshold of FOM plus score was chosen according to the best classification.

**Figure 4:**
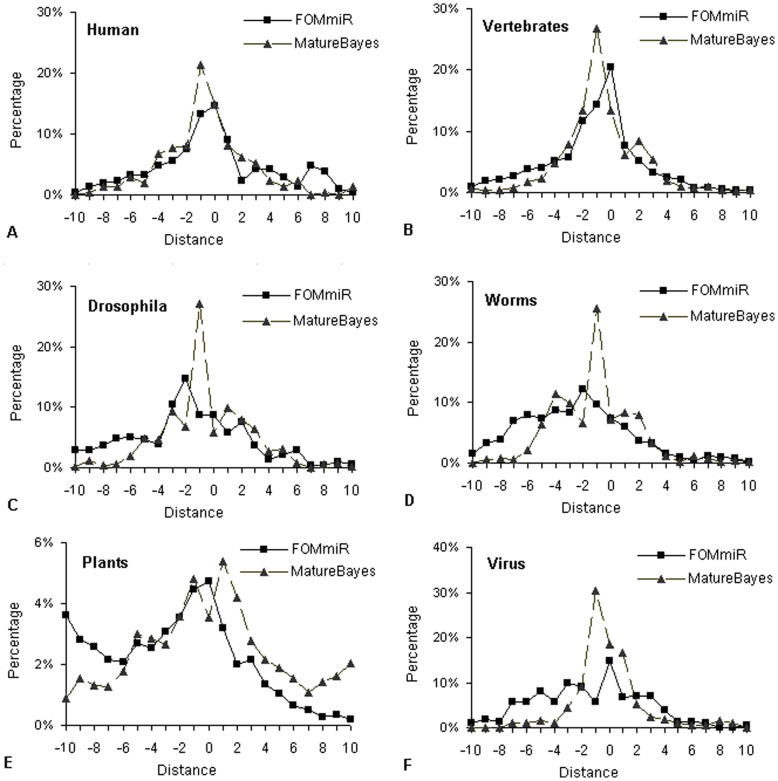
Distribution of distances between the real and predicted mature miRNA region.

#### (3) miRNA judgment

The screened segment was confirmed as the mature miRNA region, if its FOM plus score reached the threshold. Finally, the miRNA strand was figured out by the highest probability of strand emergence in the FOMmiR predictor.

**Table 3:** Quantitative distribution of miRNA strands in positive training dataset.

		Predicted		
	Strand	*5p*	*3p*	*both*
**True**	***5p***	124	68	25
	***3p***	0	269	7
	***both***	0	40	207

### Assessment of Prediction System

Several indexes were used to assess the performance of the model: Sensitivity (*Sen*), Specificity (Spe) [7], and Accuracy (*Acc*). Average sensitivity was measured by 5-fold cross-validation on a positive dataset.
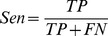


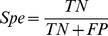


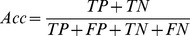



## Results

### Evaluation of the Model Parameters

As described in Methods, we designed a fixed-order Markov model for miRNA identification, known as FOMmiR. Firstly, the fixed context’s length D was been estimated based on the performance of positive and negative signal discrimination. The third order (D = 3) was much stable and chosen into FOMmiR predictor due to a consistent classification between the training and test dataset. Although the third-order Markov model achieved a satisfactory performance with independent FOM score (Figure 2A), the three MFE-related features were added as FOM plus score in order to improve the accuracy in certain level (Figure 2B). Then, Receiver operating characteristic curve (ROC) was drawn for threshold judgment (Figure 3). While the threshold value set to 11, the predictor got the best classification in the training dataset with 91.47% sensitivity (Sen), 91.07% specificity (Spe) and 91.11% accuracy (Acc).

**Table 4:** Quantitative distribution of miRNA strands in positive test dataset.

		Predicted		
	Strand	5p	3p	both
**True**	**5p**	14	45	17
	**3p**	0	54	15
	**both**	1	40	26

### Evaluation of the Model Stability Using Five-fold Cross Validation

In order to examine the stability of FOMmiR predictor, the classification performance was evaluated with 5-fold cross validation. The result showed that the FOMmiR still achieved a high performance with 91.47±2.52% sensitivity, 90.84±0.59% specificity and 90.91±0.70% accuracy, and which was very close to the above performance in the whole training dataset. Therefore, it suggested that this predictor was quite stable.

### Performance of miRNA Precursor Classification

To evaluate the performance of pre-miRNA classification, a comparative test was performed among different approaches against the independent test dataset composed of 273 real pre-miRNAs and 2807 pseudo pre-miRNAs. Despite some approaches not available any longer, we were fortunate to compare FOMmiR with Triplet-SVM [6], MiPred [13], CID-miRNA [15] and CSHMM [17], among which only CSHMM could be retrained with D1 and D2 dataset. Although the different training conditions of other three approaches might bring some small deviations to the comparative evaluation, at least the result displays that FOMmiR was able to achieve a satisfactory prediction as well as them, or even better (Table 1). Furthermore, the FOMmiR not only identified the real pre-miRNA, but also indicate the mature miRNA position that the others were unable to do. Given these, the FOMmiR exhibited an outstanding discriminatory power.

### Performance of Cross-species Classification

Cross-species performance is very important for a model trained in known species to predict new species, so it requests the model should hold a common structure feature for miRNA recognition. Here we tested the FOMmiR prediction rate in four species with the parameters trained only on human dataset. Moreover, a comparative test were performed with the four well-known approaches. The result showed that FOMmiR kept high sensitivities in the specie of vertebrates, worms and viruses, even plants (Table 2). It suggested that the FOMmiR model was reliable across species, and the FOMmiR parameters contained the basic recognition feature of the miRNA.

### Performance of Locating Mature miRNA Region

At the same time as the FOMmiR identified the pre-miRNA from the candidate hairpins, the mature miRNA region had been located. we compared its localization performance with that of MatureBayes. The MatureBayes uses naive Bayes algorithm to identify the mature miRNA from pre-miRNA, which has significantly better performance than the two existing approaches with same function, ProMiR and BayesMiRNAfind [18]. For a large number of random test set from different species, the comparative test was performed between FOMmiR and MatureBayes. The distances were calculated between the actual mature miRNA and the predicted mature miRNA. It was obvious in Figure 4 that the localization performance of FOMmiR was close to that of MatureBayes.

### Performance of Identifying Mature miRNA Strand

Identifying the mature miRNA strand from the complementary strands seems to be very difficult, few of approaches was reported to conquer it. Nevertheless, in this study, we extracted the strand information where the mature miRNA located and defined it as another FOM parameter, so the miRNA stand could be checked out from the miRNA region. The result displayed that the strand-check accuracy reached to 86.5% in positive training dataset (Table 3) and 63.7% in the positive test dataset (Table 4).

## Discussion and Conclusions

In recent years, a lot of algorithms and models have been tried to predict pre-miRNA or mature miRNA. The excellent ones are always concerned about, not only because it could predict new miRNA, but also because it might interpret the enzyme cleavage mechanism. Although the single-function prediction seems to be close to success, new generation of full-function prediction is very expected.

From the view of system biology, the biological processes always employ the parsimony principle to obtain the best energy efficiency rate. So we suppose that both Drosha/DGCR8 complex and Dicer might focus on a similar structure pattern of miRNA region, despite two independent biological processes needed for the final maturation of miRNA. In this study, we start to find the discriminatory signals in the mature miRNA regions, where the primary structure, secondary structure and their combination have been analyzed respectively in the fixed-order Markov model. But only the pure secondary structure could exhibit the significant signals. It reflects that enzyme recognition is mainly based on the secondary structure.

Based on the secondary structure pattern in the style of stem-bulge-gap notation, the FOMmiR predictor has been built using a fixed-order Markov model and successfully realized a full-function recognition of mature miRNA directly from the hairpins with similar stem-loops. All of the tests displayed that no matter on the classification of precursors, the localization of mature miRNA regions or on the cross-species ability, this approach achieves satisfactory performances in contrast to those well-known ones. Moreover, the FOMmiR experienced a successful trial in identifying the mature miRNA strand, although this function remains to improve.

The secondary structure of pre-miRNAs in plants seems much more complex than those in other species, due to more stem-branches existed in plants. Here we generally focus on the longest stem as the main stem in order to decrease the noise from those stem-branches, so the FOMmiR significantly increased the performance in plants than other algorithms did. On the other hand, with human-trained parameter, both of the FOMmiR and other algorithms obtained a similar result, in which the sensitivity in vertebrates is less than those in Drosophila and worm, even plants (Table 2).

With regard to the model construction, the FOMmiR, as one of machine learning approaches, is much simpler than those of the hidden Markov model, the stochastic context free grammar model and the support vector machine-based methods. Although the actual processes of the biological recognition are obscure, we have got a sense of the potential mechanism during the model construction. Briefly, the quadruple codes on the secondary structure pattern are quite crucial for the miRNA recognition.

Overall, in this study, we provide a new generation of miRNA prediction algorithm, using a fixed-order Markov model based on the secondary structure pattern, which successfully realizes a full-function recognition of the mature miRNAs directly from the hairpin RNA molecules.
